# Eating behaviour phenotype in the MYT1L-related neurodevelopmental disorder: a deep phenotyping study using standardized questionnaires

**DOI:** 10.1186/s13023-026-04497-x

**Published:** 2026-07-21

**Authors:** Juliette Coursimault, Emilie Guillon, François Lecoquierre, Camille Charbonnier, Anne-Marie Guerrot, Alice Goldenberg, Gaël Nicolas, Elise Schaefer, Anael Ayrolles, Richard Delorme, Florence Riccardi, Maude Grelet, Roseline Caumes, Mathilde Nizon, Bertrand Isidor, Guillaume Jouret, Caroline Rooryck, Jeanne Amiel, Anne-Sophie Alaix, Victor Morel, Adeline Jacquinet, Cyril Mignot, Laurence Faivre, Emilie Fraile-Caietta, Sarah Chalopin, Béatrice Dubern, Christine Poitou

**Affiliations:** 1 Department of Genetics and Reference Center for Developmental Disorders, Univ Rouen Normandie, Normandie Univ, Inserm U1245 and CHU Rouen, Rouen, F-76000 France; 2 Nutrition Department, Reference Center for Rare Diseases PRADORT, Assistance Publique Hôpitaux de Paris, Pitié-Salpêtrière Hospital, Paris, France; 3 Sorbonne Université, Inserm, Nutrition and Obesities: Systemic Approaches, NutriOmique Research Group, Paris, France; 4 Service de Génétique Médicale, Institut de Génétique Médicale d’Alsace (IGMA), Hôpitaux Universitaires de Strasbourg, Strasbourg, France; 5 Department of Child and Adolescent Psychiatry, APHP.Nord, Robert Debré University Hospital, Paris Cité University, Paris, France; 6 Génétique Médicale, Centre Hospitalier Intercommunal Toulon - La Seyne- sur-Mer, Toulon, France; 7 Université de Lille, CHU de Lille, Clinique de Génétique «Guy Fontaine», EA7364 RADEMEF-59000, Lille, France; 8 Service de Génétique Médicale, CHU Nantes, Nantes, France; 9 National Center of Genetics (NCG), Laboratoire National de Santé (LNS), Dudelange, L- 3555 Luxembourg; 10 Univ. Bordeaux, MRGM INSERM U1211, CHU de Bordeaux, Service de Génétique Médicale, Bordeaux, F-33000 France; 11 Service de Médecine Génomique des Maladies Rares, Hôpital Necker- Enfants Malades, AP-HP, Paris, France; 12 Department of Medical Genetics, La Timone Hospital, AP-HM, La Timone Children’s Hospital, Marseille, France; 13 Department of Genetics, Sart Tilman University Hospital, Liège, Belgium; 14 Département de Génétique, Groupe Hospitalier Pitié-Salpêtrière-Hôpital Trousseau Centre de Référence Déficiences Intellectuelles de Causes Rares, APHP.Sorbonne Université, Paris, France; 15 Université Bourgogne Europe, CHU Dijon Bourgogne, Inserm, CTM UMR1231, équipe GAD, FHU TRANSLAD, Centre de génétique, Centre de référence Anomalies du Développement et Syndromes Malformatifs, Centre de référence Déficiences Intellectuelles de Causes Rares, et Centre de référence GénoPsy, Dijon, 21000 France; 16 Service de pédiatrie, Centre Hospitalier de Toulon, Toulon, France; 17 Nutrition and Gastroenterology Department, Reference Center for Rare Diseases PRADORT, Assistance Publique Hôpitaux de Paris, Trousseau Hospital, Paris, France

**Keywords:** *MYT1L* haploinsufficiency, 2p25.3 deletion, Deep phenotypic characterization, Standardized questionnaires, Syndromic obesity, Eating behaviours, Rare disease, Dykens

## Abstract

**Background:**

The MYT1L-related neurodevelopmental disorder (MRND) is associated with global motor and language delay, intellectual disability, behavioural disturbances, epilepsy and frequent early-onset obesity. Eating disturbances have been reported but remain poorly characterized. A systematic characterization of the eating behaviour phenotype is essential to improve diagnosis and management.

**Methods:**

We conducted a multicentre study including 21 French-speaking individuals with a molecularly confirmed MRND and either overweight/obesity or eating behaviour disturbances. Eating behaviours were reported by relatives using validated questionnaires widely employed in rare obesity research: the Dykens Hyperphagia Questionnaire (HQ), the Trousseau Impulsivity Questionnaire, the Children’s Eating Behaviour Questionnaire (CEBQ), the Food Cravings Questionnaire-Trait-reduced (FCQ-T-r, adults only), and a Visual Analogue Scale (VAS) for hunger. When available, questionnaire results were compared with published studies using the same instruments.

**Results:**

Persistent hyperphagic behaviours were identified in the majority of patients, with the HQ questionnaire highlighting sustained food-seeking behaviours and pervasive food preoccupation. The mean global score was 25.9, indicating a moderate yet sustained hyperphagic profile. CEBQ revealed a strong attraction to food combined with reduced satiety responsiveness and rapid eating. Hunger was reported as persistent throughout the day. In children food impulsivity was less frequent (5/14; 36%) and no correlation was found between impulsivity and hyperphagia severity, contrasting with findings in other obesity syndromes. Among adults, 62.5% had scores suggestive of food addiction (FCQ-T-*r* ≥ 50).

**Conclusion:**

This study delineates an eating behaviour profile in a symptom-enriched subgroup of individuals with MRND, characterized by moderate but sustained hyperphagia, persistent hunger, impaired satiety, low food-related impulsivity in children but frequent food craving/addiction in adults. These findings support early, age-adapted and multidisciplinary management strategies and highlight the need for targeted interventions to reduce the burden on affected individuals and families.

**Supplementary Information:**

The online version contains supplementary material available at https://doi.org/10.1186/s13023-026-04497-x.

## Background

Obesity and overweight occur more frequently in individuals with intellectual developmental disorders than in the general population, with prevalence rates ranging from 22% to 38% depending on age and setting [14, 16, 19]. Increased risk has been attributed to a combination of environmental and genetic factors. Environmental contributors include inequality in access to healthcare, limited opportunities for screening and prevention, reduced access to adapted physical activity, communication difficulties, adverse effects of psychotropic medications, and insufficient training among healthcare professionals. On the genetic side, more than 50 syndromes have been identified as predisposing to early-onset obesity [7], including Prader–Willi syndrome (PWS), Bardet–Biedl syndrome (BBS), fragile X syndrome, and 16p11.2 deletion. In many of them, specific eating behaviour phenotypes—such as hyperphagia, food preoccupation, impaired satiety, or impulsivity—are central drivers of excess weight gain and represent major challenges for families and clinicians.

The MYT1L-related neurodevelopmental disorder (MRND) is a recently described rare condition caused by haploinsufficiency of the *MYT1L* gene due to either 2p25.3 deletions or deleterious point mutations. Core features include motor and language delays, hypotonia, intellectual disability, behavioural disturbances, and distinctive dysmorphic traits [3, 6, 25, 30]. In addition, obesity and eating behaviour disturbances are reported in approximately 60% and 45% of patients, respectively, with a median age at onset of weight gain around three years [6]. The feeding behavioural spectrum is broad, ranging from persistent food preoccupation and reduced satiety to impulsivity and rapid eating. However, available data remain fragmentary based on small series and anecdotal descriptions. To date, no systematic characterization of eating behaviour has yet been performed in individuals with MRND presenting with overweight/obesity or eating behaviour disturbances, and no genotype–phenotype correlation has been established.

Experimental models have provided insights into the possible biological underpinnings of obesity in *MYT1L* haploinsufficiency. In mice, Myt1l knockout leads to lifelong obesity, more pronounced in females, without major endocrine abnormalities or reduced lifespan [5, 24, 31]. In zebrafish, Myt1l knockdown results in reduced oxytocin (OXT) and vasopressin expression in hypothalamic homologues, suggesting impaired development of feeding-related neuroendocrine circuits [3]. More recently, mouse models revealed divergent findings, with decreased AVP but increased OXT cell numbers in the paraventricular nucleus (PVN), and largely preserved hypothalamic mediated behaviours [20]. While these data point to a role for *MYT1L* in the leptin–melanocortin–*SIM1* pathway, no consistent metabolic signature has been identified, reinforcing the need for detailed clinical phenotyping.

The clinical burden of eating disorders in rare obesity syndromes is considerable. In PWS, for instance, hyperphagia is a hallmark symptom and the main determinant of caregiver burden, shaping both daily routines and long-term prognosis [15]. To date, no data are available on quality of life (QoL) in MRND, either in relation to broader behavioural difficulties specific to the condition (e.g., frustration intolerance, anxiety, aggressiveness, autism) or to eating disturbances. Similar but less severe profiles have been reported in BBS and other syndromes, suggesting that precise phenotyping of eating behaviour can help position each disorder along a continuum of severity [1, 12, 29, 32]. Such phenotyping has proven crucial for adapting management strategies, from behavioural interventions to the design of clinical trials targeting appetite regulation. In the context of MRND, the contribution of eating behaviour disturbances to obesity remains underexplored. A clearer delineation is essential to guide both patient management and future pharmacological trials.

In this study, we aimed to provide the first detailed characterization of eating behaviours in children and adults with MYT1L-related neurodevelopmental disorder presenting with overweight/obesity or eating behaviour disturbances. We used standardized questionnaires widely used not only in rare obesity research but also in clinical practice, as they are recommended in the French national care pathway for rare obesities (PNDS). Our objectives were to (i) improve the delineation of the eating behaviour profile of this syndrome, (ii) compare it with published data from other rare obesities, and (iii) discuss clinical implications for early diagnosis and management.

## Methods

### Study population

We recruited French-speaking patients with a molecularly confirmed MRND, either due to a pathogenic/likely pathogenic variant or a *de novo* deletion encompassing the *MYT1L* gene. Inclusion criteria were a confirmed molecular diagnosis and the presence of either overweight/obesity or clinically reported eating behaviour disturbances, in order to specifically investigate eating-related phenotypes. Overweight and obesity were defined according to World Health Organization (WHO) standards: in children, BMI-for-age > + 1 SD (overweight) or > + 2 SD (obesity); in adults, BMI 25.0–29.9 kg/m² (overweight) or ≥ 30.0 kg/m² (obesity). Questionnaires were completed and submitted by families or their referring clinicians as part of their routine care. The study was approved by the institutional ethics committee of Rouen University Hospital (notification n°69–2020).

### Eating behaviour assessment

Eating behaviours were evaluated using validated questionnaires widely employed both in rare obesity research and in clinical practice (Supplementary Table 1):

- Dykens Hyperphagia Questionnaire (HQ) [8]: 11 items scored on behaviour, drive, and severity, with a total score ranging from 11 to 55, assessing food-centred thinking, food seeking and stealing behaviour. In the French national diagnostic and care protocol (PNDS) for PWS, developed as an expert consensus, a total score of ≥ 19 on the Dykens Hyperphagia Questionnaire is considered to reflect clinically significant (marked) hyperphagia. This threshold has since been used in research on other rare obesity syndromes, although it has not been formally validated as a universal cut-off in the original questionnaire.
- Trousseau Impulsivity Questionnaire (in-house tool) [1]: 10 items assessing food-related impulsivity; a score ≥ 7 has been proposed as an exploratory clinical threshold, though it has not been formally validated in MRND.
- Children’s Eating Behaviour Questionnaire (CEBQ) [28]: a validated tool to measure hunger and satiety, food-centred thinking, food preference and ingestive behaviour in children. Although the CEBQ was primarily validated in children, it has also been applied to adults with neurodevelopmental disorders and/or intellectual disability, as supported by a recent systematic review [12]. 35 items covering eight domains, grouped into food-approach (Food Responsiveness, Emotional Overeating, Enjoyment of Food, Desire for Drinks) and food-avoidant (Satiety Responsiveness, Slowness in Eating, Emotional Undereating, Food Fussiness) behaviours. Scores range from 1 (never) to 5 (always).
- Food Cravings Questionnaire–Trait reduced (FCQ-T-r) [4, 21, 22]: reduced version of 15 items, rated on a 6-point scale (1 = never to 6 = always), yielding a total score between 15 and 90, assessing food craving and addiction in adults. Scores ≥ 50 are suggestive of food addiction.
- Visual Analogue Scale (VAS) for hunger [10]: four items assessing average, peak, minimal, and morning hunger over the preceding 24 h, based on caregiver-supported reporting when needed (0 = not at all hungry, 10 = extremely hungry).
- Parental-Developmental Disorders-Quality of Life (PAR-DD-QoL) [2, 23]: a 17-item self-report scale (5-point Likert) yielding emotional, adaptive, and global scores (0–100), with higher scores indicating greater impact on caregiver quality of life. For this study, this questionnaire was focused on quality of life linked to food behaviour.

Whenever possible, scores were compared with published data from control populations or other rare obesity syndromes (Supplementary Table 2).

### Data analysis

Descriptive statistics were reported as means, medians, and ranges. Associations between hyperphagia (HQ total score) and impulsivity were assessed using Pearson correlation coefficients and group comparisons (impulsivity ≥ 7 vs. <7).

The HQ total score of the MRND cohort was compared with previously published data from rare obesity disorders, using a two-sided Welch’s t-test.

Four control studies for the CEBQ and three for the FCQ-T-r questionnaire, each reporting summary statistics (mean, standard deviation, and sample size), were separately pooled into questionnaire-specific reference groups using using a fixed-effect inverse-variance meta-analytic model, in which each study was weighted proportionally to its sample size divided by its variance. The pooled mean and its standard error were then used as the control reference for comparison with the MYT1L cohort via a two-sided Welch’s t-test. For the CEBQ questionnaire, multiple testing correction was applied across all 8 subscores simultaneously using the false discovery rate (FDR) correction.

Corrected p-values < 0.05 were considered statistically significant.

### Supplementary assessments

As part of the overall assessment of the MRND, three additional questionnaires were used to capture determinants of weight regulation beyond eating behaviour, namely social deprivation, psychological status, and physical activity. These dimensions, although outside the primary scope of eating behaviour phenotyping, are important to explore given their potential impact on obesity risk. For this purpose, parental social deprivation (EPICES), anxiety and depression (HADS, adults only), and physical activity (RPAQ) were also collected and are reported in the Supplementary Material (Supplementary Tables 1 and 3).

## Results

### Demographic, molecular and clinical characteristics of the population

The study included 21 patients (12 females, 9 males), comprising 14 children and 7 adults (Table 1 and Supplementary Table 4). Ten were newly enrolled, while 11 had been described previously [6]. The mean age at inclusion was 16.0 years (median 10, range 6–37). At the molecular level, 16 of the 21 patients carried a loss-of-function (LOF) variant: 14 patients (67%) had a truncating MYT1L variant, while 2 patients (10%) carried a deletion encompassing the gene. Five patients (24%) carried a missense variant—always within zinc finger domains— (Supplementary Tables 2 and 5 and supplementary Fig. 1). Variants were *de novo* in 95% of cases, with inheritance unknown in one patient. Eight variants had not been previously described. Overall, this distribution was consistent with reported *MYT1L* molecular profiles [6].

All participants were included based on the presence of either overweight/obesity or parent-reported eating behaviour disturbances (see methods). 70% (14/20) of participants were persons with obesity, 15% (3/20) were persons with overweight, and 15% (3/20) had a normal weight. Accelerated weight trajectory or parental concern about weight typically began early, with a mean onset of 3 years (median = 3) (Supplementary Table 3). 76% (16/21) of participants were reported by their parents to have eating behavioural disturbances, of whom 75% (12/16) were persons with overweight or obesity.

Most patients (70%) were followed by an endocrinologist or nutrition specialist, and 60% had consulted a dietitian, either occasionally or within a multidisciplinary program. Compared with global data showing that 40–70% of children with obesity have at least one obese parent [17], 38% of parents in our cohort were reported as overweight or obese and no sibling obesity was reported. Family social deprivation was similar in our cohort (28.5% with an EPICES score > 30) to that observed in a PWS family cohort (25.9%; [11]).

Concerning physical activity, fourteen children and six adults completed the RPAQ (Supplementary Table 3). Children engaged in an average of 71 min/day of light activity and 433 min/week of moderate activity, mainly walking and cycling, with limited vigorous activity (59 min/week). Adults reported lower light activity (20 min/day) but similar levels of moderate activity (≈ 500 min/week, mostly walking), and rare vigorous activity (26 min/week). Both children and adults reported low frequency of structured physical training.

Half of the cohort (11/21) were receiving pharmacological treatments for MRND-related manifestations such as behavioural symptoms or epilepsy (supplementary Table 6). Several of these medications may influence appetite or weight, either through appetite reduction and potential weight loss, as reported for methylphenidate, atomoxetine, and fluoxetine, or through increased appetite and weight gain, as reported for valproate, aripiprazole, and alimemazine. Other treatments had more variable or neutral expected effects on weight. Ten participants were receiving medications known to influence appetite and weight (Patient 18, who was taking a poppy-based dietary supplement at the time of questionnaire completion rather than a pharmacological treatment was excluded from statistical analyses). One patient was receiving a medication potentially associated with weight gain at the time of questionnaire completion, five patients were receiving one or more medications potentially associated with reduced weight gain, two patients were receiving medications considered to have variable effects on weight, and two patients were receiving combinations of medications with both weight-promoting and weight-reducing effects. No significant differences were observed in questionnaire scores between medicated and non-medicated patients (excluding patient 18, as above). Due to small and heterogeneous subgroups, more detailed stratified analyses were not feasible. According to parental reports, several treatments were associated with weight gain (valproate, methylphenidate) or behavioural worsening (aripiprazole, risperidone, methylphenidate), whereas others, such as methylphenidate and atomoxetine, were perceived as partially beneficial for appetite control. Only one patient had received semaglutide (1 mg/week) with temporary benefit.

HADS scores in adults indicated no clinically depression (mean score = 7.1) or anxiety (mean score = 6.6), with four borderline case but no pathological values.

**Table 1:** Demographic characteristics of the study population

	*n*	total	%
**Index Case Characteristics**			
***Sex***			
Female	12	21	57%
Male	9	21	43%
*** Age at inclusion***			
< 18 years old	14	21	67%
≥ 18 years old	7	21	33%
*** Variant type***			
Truncating	14	21	67%
Missense	5	21	24%
Deletion	2	21	10%
*** Inheritance***			
De novo	20	21	95%
Unkown	1	21	5%
*** Weight***			
Standard Weight	3	20	15%
Overweight	3	20	15%
Obesity	14	20	70%
*** Age at weight gain***			
< 6yo	16	21	76%
6–15 yo	5	21	24%
≥ 15 yo	0	21	0%
*** Reported to have eating behaviour disturbance (by parents)***			
Eating disturbances reported	16	21	76%
No eating disturbances reported	5	21	24%
*** Endocrinologist follow-up***			
Followed-up	14	20	70%
No follow-up	6	20	30%
*** Dietetician follow-up***			
Followed-up	12	20	60%
No follow-up	8	20	40%
*** Family history***			
Parental history of overweight or obesity	8	21	38%
Siblings history of overweight or obesity	0	18	0%
*** History of treatments***			
Regarding the *MYT1L*-related disorder	11	21	52%
Regarding obesity management	1	21	5%

For each variable, the number of affected individuals, the total number, and the corresponding percentage are reported. Yo: years old

### Eating behaviour characteristics of patients

#### VAS for hunger

Among 19 patients (including 7 adults), the mean perceived hunger scores over the past 24 h were 5.1 for average hunger, 5.7 for peak hunger, 2.5 for lowest hunger and 5.3 for hunger upon waking. These findings indicate, over the preceding 24 h, persistent hunger throughout the day, including upon waking with minimal relief even at the lowest levels.

#### The Dykens’ Hyperphagia Questionnaire (HQ)

**Fig. 1:**
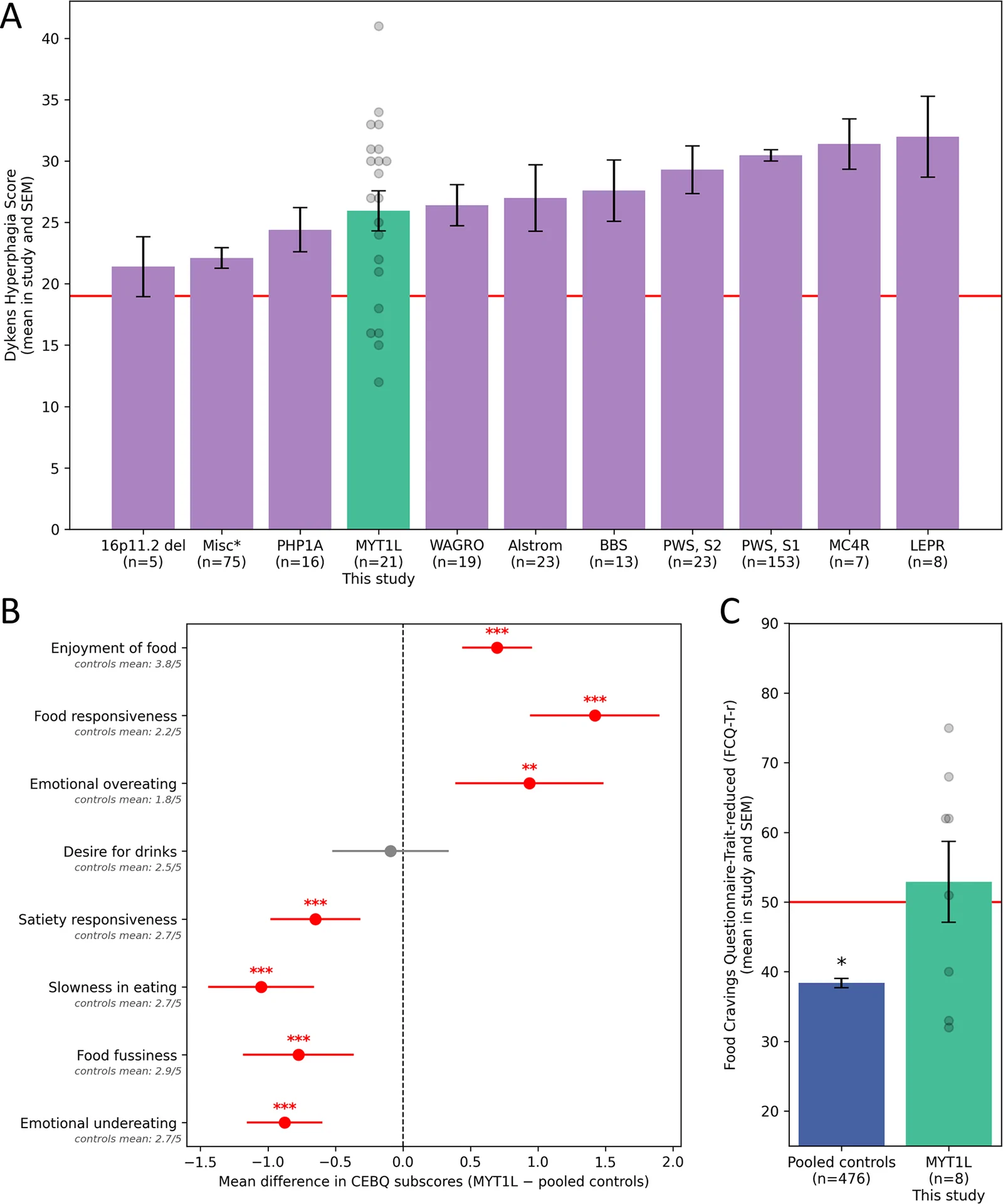
Eating behaviour profiles in MYT1L patients compared with literature cohorts. Individual data points are shown for the present study; literature data are displayed as means. Bars indicate mean ± SEM. Stars denote FDR-corrected p-values (*p < 0.05, **p < 0.01, ***p < 0.001; correction applied within each subscale). **(A)** Global Dykens Hyperphagia Questionnaire (HQ) scores in MYT1L compared with rare genetic obesity cohorts. The HQ ranges from 11–55; the red line indicates the clinical threshold for hyperphagia (≥ 19). Purple indicates patients with rare obesity disorders reported in the literature, whereas green indicates MYT1L patients. Misc* stands for miscellaneous including patients with several distinct etiologies (hypothalamic obesity, ID with obesity, polygenic obesity; Arnouk 2024). **(B)** Forest plot of CEBQ scores in MYT1L compared with non-affected children from several countries (UK, Spain, Switzerland and Portugal), pooled in one control group. CEBQ scores range from 1 to 5, with higher values indicating stronger behaviours. **(C)** FCQ-T-r total scores in adults with MYT1L syndrome compared with a pooled control cohort based on published data from German students and Iranian male and female student samples [22, 26]. Participants with MYT1L syndrome are displayed in green and controls in blue. Scores range from 15–90; the red line indicates the threshold for food addiction (≥ 50)

Among the 21 patients, the mean Dykens’ total score was 25.9 (median 27, range 12–41). A total Dykens’ score ≥ 19, regarding the French threshold, was observed in 16 individuals, representing 76% of the cohort. The subscores indicated moderate behavioural disturbance (mean 9.9/25), elevated drive (11.5/20), and moderate severity (4.6/10). Compared published data from other rare obesity syndromes, patients with MRND exhibited a moderate hyperphagia profile, with a mean HQ scores within the range reported in Prader–Willi syndrome (30.5 in Dykens et al. and 29.3 in Hollander et al.) and Bardet–Biedl syndrome (27.6 in Sherafat-Kazemzadeh et al.), higher than in 16p11.2 deletion (21.4 in Zorn et al.), but lower than in monoallelic MC4R and biallelic LEPR deficiencies (31.4 and 32, respectively, in Zorn et al.) (Supplementary Table 2; Fig. 1A)). Two groups differed significantly when considered individually — Misc* and PW S1 — due to the large sample sizes, but these differences were no longer significant after correction for multiple testing (Supplementary Table 2). The first signs of interest in food appeared at a mean age of 3.5 years (median 3; range 0–7), with five parents reporting concerns before the first year of life. In most cases (63%), preoccupation with food was reported as stable over time.

#### The Children’s Eating Behaviour Questionnaire (CEBQ)

The CEBQ, completed for 21 individuals showed a pattern of increased elevated food-approach behaviours and reduced self-regulation. The highest mean subscale scores were observed for Enjoyment of Food (4.5/5) and Food Responsiveness (3.7/5), while Satiety Responsiveness (2.1/5), Food Fussiness (2.1/5) Slowness in Eating (1.7/5) were the lowest. Emotional Overeating (2.7/5) and Emotional Undereating (1.8/5) were moderate to low, Desire to Drink subscale (2.4/5) showed moderate scores (Fig. 1B). Compared with pooled normative data from unaffected children from multiple countries (UK, Spain, Switzerland and Portugal, Supplementary Table 2) [9, 18, 27, 28], persons with MYT1L syndrome scored significantly higher on Enjoyment of Food and Food responsiveness suggesting greater enjoyment of eating and increased responsiveness to food stimuli (Fig. 1B). They scored significantly lower on all food-avoidant subscales suggesting reduced sensitivity to satiety, faster eating pace, broad food acceptance and less influence of negative emotions on appetite. The MYT1L group exhibited moderately elevated Emotional Overeating scores compared with the control cohort, reaching statistical significance after correction. Desire for drinks did not differ significantly between groups.

#### The Food Cravings Questionnaire-Trait-reduced (FCQ-T-r)

Eight patients (seven adults and one 17-year-old adolescent) completed the FCQ-T-r with caregiver support. The mean score for the cohort was 52.9 ± 7.5 (SD), with 5/8 participants (62.5%) scoring above threshold suggestive of food addiction (≥ 50) (Fig. 1C). This mean score was significantly higher than those reported in published control populations, including student samples from Germany (mean 38.5) and Iran (mean 38.4 in females and 37.6 in males; Supplementary Table 2 [22, 26].

#### Impulsivity Questionnaire (Trousseau in-house questionnaire)

Eighteen patients, including 14 children, completed the in-house impulsivity questionnaire. Five of 18 individuals (28%) had a score ≥ 7 (mean 5.2, median 6), including 5 of 14 children (36%). Additional items revealed difficulty adapting to unexpected situations (78%), frequent preoccupation with food outside mealtimes (44%), while no cases of night-time food seeking were reported. Unlike Arnouk et al., [1], who reported an association between impulsivity and hyperphagia in other obesity groups, no such correlation was found in our cohort (*p* > 0.05, Supplementary Fig. 2).

#### PAR-DD-Qol questionnaire, adapted to eating behaviour

Twenty parents completed the PAR-DD-QoL. The mean emotional subscore was 24.5, and the adaptive subscore 15.9, yielding a global score of 40.4. Overall, 63% reported a slightly to moderately degraded quality of life and 37% a severely or extremely degraded quality of life.

#### Genotype-phenotype correlation

No significant differences were found in questionnaire responses between LOF variants (truncating and deletions) and missense variants after Bonferroni correction; however, these results should be interpreted with caution given the small sample size (Supplementary Table 7).

## Discussion

In this study, we specifically characterised eating behaviours in individuals with MRND presenting with overweight/obesity and/or eating behaviour disturbances, thereby capturing a clinically enriched subgroup in which metabolic and behavioural manifestations may be particularly apparent. Our results highlight a complex profile that combines early-onset hyperphagia, impaired satiety, persistent food-centred thinking, situational food seeking, moderate impulsivity, pronounced food craving in adults, and frequent oral-motor difficulties. We also provide novel data on quality of life, physical activity, and socioeconomic context, which together illustrate the multidimensional burden of this condition.

### Early-onset weight gain and endocrine profile in MRND

Early-onset weight gain in MRND is consistent with previous reports (median onset 3 years), placing the condition within the earliest-onset group of rare obesities, alongside PWS and BBS [6]. Unlike in PWS, where endocrine deficiencies often coexist and contribute to metabolic complications, endocrine abnormalities were not a prominent feature in our sample. The combination of early excessive weight gain, neurodevelopmental impairment, and normal endocrine assessments should therefore be considered a clinical red flag for *MYT1L* haploinsufficiency.

### Hyperphagia

Hyperphagia, defined as excessive food intake, heightened hunger, reduced satiety, persistent food-seeking behaviours, and associated psychological distress [13] emerged as one of the most frequent and impactful symptoms in our cohort. The Dykens questionnaire highlighted moderate but persistent hyperphagia, with subscores reflecting both perseverance in food seeking and a pervasive preoccupation with food. The onset of abnormal appetite typically occurred before six years of age, sometimes in infancy. This timing places MRND within the group of early-onset hyperphagic disorders, alongside PWS and BBS, in contrast with conditions such as fragile X or Angelman syndrome where weight gain tends to occur later [7].

Compared with other rare genetic obesities, MYT1L-related hyperphagia appears less severe and disruptive than in MC4R and LEPR deficiencies, where hyperphagia is often extreme and resistant to behavioural control. It is closer in intensity to PWS or BBS. Unlike PWS, where hyperphagia progressively worsens through childhood and adolescence, food preoccupation in MYT1L patients was generally reported as stable over time, suggesting a distinct natural history. As such, the present findings should be interpreted within a symptom-enriched cohort.

### Satiety regulation and persistent hunger

Impaired satiety was consistently reported, with low CEBQ satiety responsiveness and persistently elevated hunger scores on the VAS, based on data reported over the preceding 24 h. Patients described hunger present upon waking and rarely relieved even at the lowest levels. These findings echo observations in leptin–melanocortin pathway disorders but seem less extreme than in PWS, where satiety is virtually absent. Tachyphagia, chewing difficulties, and swallowing of large pieces—reported in over two thirds of patients—likely compound satiety problems by accelerating ingestion and reducing oro-sensory feedback. Such oral-motor issues, rarely highlighted in other syndromes, may represent a distinctive feature of MYT1L-related obesity.

### Food-centred thinking and food seeking behaviours

A pervasive cognitive preoccupation with food was supported by multiple questionnaires: elevated Dykens’ drive/severity subscores, high CEBQ enjoyment of food and food responsiveness, and elevated adult craving scores. Half of the patients displayed active food-seeking behaviours such as stealing food or persevering in its pursuit. However, night-time food seeking—typical of PWS—was absent, suggesting a less compulsive and less environmentally disruptive pattern. Compared with BBS, the MYT1L phenotype was marked by greater food enjoyment but lower food selectivity, consistent with broad food acceptance and low fussiness.

### Impulsivity and loss of control eating

Food-related impulsivity was identified in only 28% of all patients (5/18) and showed no association with hyperphagia severity. This contrasts with the findings from other obesity group, where impulsivity reinforced hyperphagia [1]. In the MRND, impulsivity appears less central, although preoccupation with food outside meals was still frequent. In adulthood, however, food craving and loss of control became prominent: 63% of adults scored above the FCQ-T-r threshold for food addiction, with mean scores substantially higher than in control populations. This pattern suggests an age-related evolution of the eating phenotype, with craving and loss of control eating emerging later, and may have important consequences for long-term management.

### Impact of medication on questionnaires outcomes

Ten participants were receiving pharmacological treatments at the time of the study with known or potential effects on appetite and weight, including medications associated with weight gain, weight reduction, neutral effects, or mixed profiles. Although only a small number of patients were exposed to each category, this heterogeneity highlights a potential source of confounding in the interpretation of questionnaire outcomes. Indeed, these pharmacological treatments may have modulated patients’ behaviour, thereby indirectly influencing parental perceptions and responses to the questionnaires. However, given the limited sample size and the diversity of treatments, the study was not sufficiently powered to formally assess these effects. Consequently, our findings should be interpreted with caution, as residual confounding related to medication exposure cannot be excluded.

### Familial quality of life and social deprivation

Caregivers of individuals with MRND reported a substantial burden, with 63% indicating a slight to moderate deterioration in quality of life and 37% reporting severe or extreme deterioration. The median PAR-DD-QoL score (42) was similar to that observed in parents of children with autism spectrum disorders or severe chronic diseases (46.7 and 44.7, respectively) [2]. This variability highlights the need for individualized family support. Social deprivation was also frequent, with 28.5% of families scoring above the EPICES threshold, comparable to PWS cohorts (25.9%), underscoring the importance of early identification of vulnerable families and the implementation of family-centered strategies [11].

### Physical activities

This study provides a description of the impact of physical activity and sedentary in individuals with MRND. Children were generally more active than adults, engaging in activities such as cycling and swimming, whereas adults fell short of WHO recommendations and mainly relied on walking. The scarcity of structured, vigorous, and muscle-strengthening activities, together with high screen time, underscores the need for targeted interventions, in the context of a neurodevelopmental disorder where barriers to physical activity are numerous (motor difficulties, hypotonia, fatigue, behavioural issues, and limited access to adapted programs). Without targeted interventions, reduced physical activity reinforce sedentary habits, weight gain, and social withdrawal, thereby perpetuating a downward spiral.

### Limitations of the study

Our study has several limitations that should be acknowledged. First, the small sample size (21 participants) reflects the rarity of the condition and recruitment challenges, and limits statistical power and generalizability. The presence of atypical cases may also disproportionately influence group averages. Only francophone patients were included, as validated translations of certain questionnaires were unavailable in other languages, further restricting external validity. Second, the study population was selected on the basis of overweight/obesity or eating behaviour disturbances and therefore represents a symptom-enriched subgroup rather than the full phenotypic spectrum of MRND. Individuals without weight problems or overt food-related difficulties were not included, which may have led to an overestimation of the prevalence and severity of eating-related behaviours and limits generalizability. Third, the study design captured a single time-point snapshot, limiting our ability to assess temporal variability. While the VAS provided a partial view of daily fluctuations in hunger, as it was based on data reported over the preceding 24 h, longitudinal data would be required to evaluate changes across developmental stages or in response to interventions. Fourth, reliance on caregiver-reported questionnaires introduces the risk of recall bias and subjective interpretation. This was particularly relevant for retrospective items such as age at onset of weight gain, which sometimes diverged between parental reports and interview data. Misunderstanding of some items may also have influenced responses, and when both parents answered, averaging divergent responses could artificially reduce variability. Fifth, the age distribution was skewed towards children, with few adults included. This limits extrapolation to adult phenotypes, especially regarding loss of control eating and food addiction, which may emerge later in life. Voluntary participation may also have introduced selection bias, with more engaged or motivated families more likely to participate. Finally, several tool-specific limitations must be noted. The impulsivity threshold used for the Trousseau questionnaire is exploratory and not validated outside obesity cohorts. Therefore, the classification of moderate impulsivity in this cohort, based on this tool, should be interpreted with caution. Replication in independant cohorts would be valuable. The CEBQ is validated in children but not in adults, yet it was applied across the full cohort. Missing data in the VAS (only 19 participants), together with difficulties encountered by younger participants in understanding and using the scale, may have introduced measurement variability and should be considered when interpreting these findings. Moreover, these tools capture caregiver or self-representation of behaviours rather than direct observation. Comparisons with other syndromes were based on published means, often without individual-level data or matched controls, which reduces the robustness of cross-syndrome comparisons.

Despite these limitations, the use of standardized questionnaires remains a feasible and informative approach in rare disease research. It enables structured, cost-effective, and reproducible data collection, and generates hypotheses that can be tested in larger or longitudinal cohorts. In the context of MRND, this approach provides valuable preliminary insights into the eating behaviour phenotype, which has been largely unexplored to date.

### Clinical and translational implications

The eating phenotype observed in MRND appears to be characterised by moderate hyperphagia with persistent hunger, pervasive food-centred thinking, situational food seeking, low impulsivity, age-related craving, and frequent oral-motor difficulties.

These findings suggest several clinically relevant considerations:

1. earlier recognition of MRND when early-onset weight gain coexists with neurodevelopmental delay; even in the absence of endocrine abnormalities.
2. anticipatory counselling for families regarding persistent hunger and food preoccupation;
3. structured mealtime routines and environmental adaptations;
4. attention to oral-motor issues such as rapid eating/tachyphagia; and.
5. age-adapted monitoring, including craving/loss-of-control eating in adults.

In summary, the MYT1L-related neurodevelopmental disorder is marked by early-onset weight gain associated with moderate but persistent hyperphagia, food preoccupation, low impulsivity in children and prominent craving in adults, and frequent oral-motor difficulties. These eating disturbances significantly affect families and highlight the need for early and proactive management. Preventive strategies implemented at the time of genetic diagnosis—such as nutritional counselling, structured mealtime routines, and promotion of adapted physical activity—may help to limit weight progression and reduce the risk of obesity-related complications, thereby avoiding the emergence of a double burden combining neurodevelopmental disability and metabolic comorbidities. A multidisciplinary and family-centred approach remains essential, ideally within specialized centres for rare diseases and complex obesity. Overall, these findings delineate an eating behaviour profile within a symptom-enriched MRND cohort and should be interpreted as characterising this clinically selected subgroup rather than providing prevalence estimates for the broader MRND population.

## Electronic Supplementary Material

Below is the link to the electronic supplementary material.

Supplementary Material 1

Supplementary Material 2

## Data Availability

The dataset used during the current study is included in this published article and is available from the corresponding author on reasonable request.

## Abbreviations

MRND: MYT1L-related neurodevelopmental disorder
PWS: Prader-Willi syndrome
BBS: Bardet-Biedl Syndrome
BMI: Body mass index
CEBQ: Child Eating Behavior Questionnaire
WHO: World Health Organization
QoL: Quality of life
RPAQ: Recent Physical Activity Questionnaire
FCQ-T-r: Food Cravings Questionnaire-Trait-reduced
HADS: Hospital Anxiety and Depression Scale
EPICES: Évaluation de la Précarité et des Inégalités de Santé dans les Centres d’Examens de Santé
PAR-DD-QoL: Parental Developmental Disabilities Quality of Life
PNDS: Protocoles Nationaux de Diagnostic et de Soins
PRADORT: Prader-Willi syndrome and obesity with eating disorders
LOF: Loss of function
