# Supplementary material for: Eating behaviour phenotype in the MYT1L-related neurodevelopmental disorder: a deep phenotyping study using standardized questionnaires
Source: Orphanet J Rare Dis. 2026 Jul 21;21:368. doi: 10.1186/s13023-026-04497-x (PMC13685170; doi:10.1186/s13023-026-04497-x)
Supplement: Supplementary file 1 [file 13023_2026_4497_MOESM1_ESM.pdf]

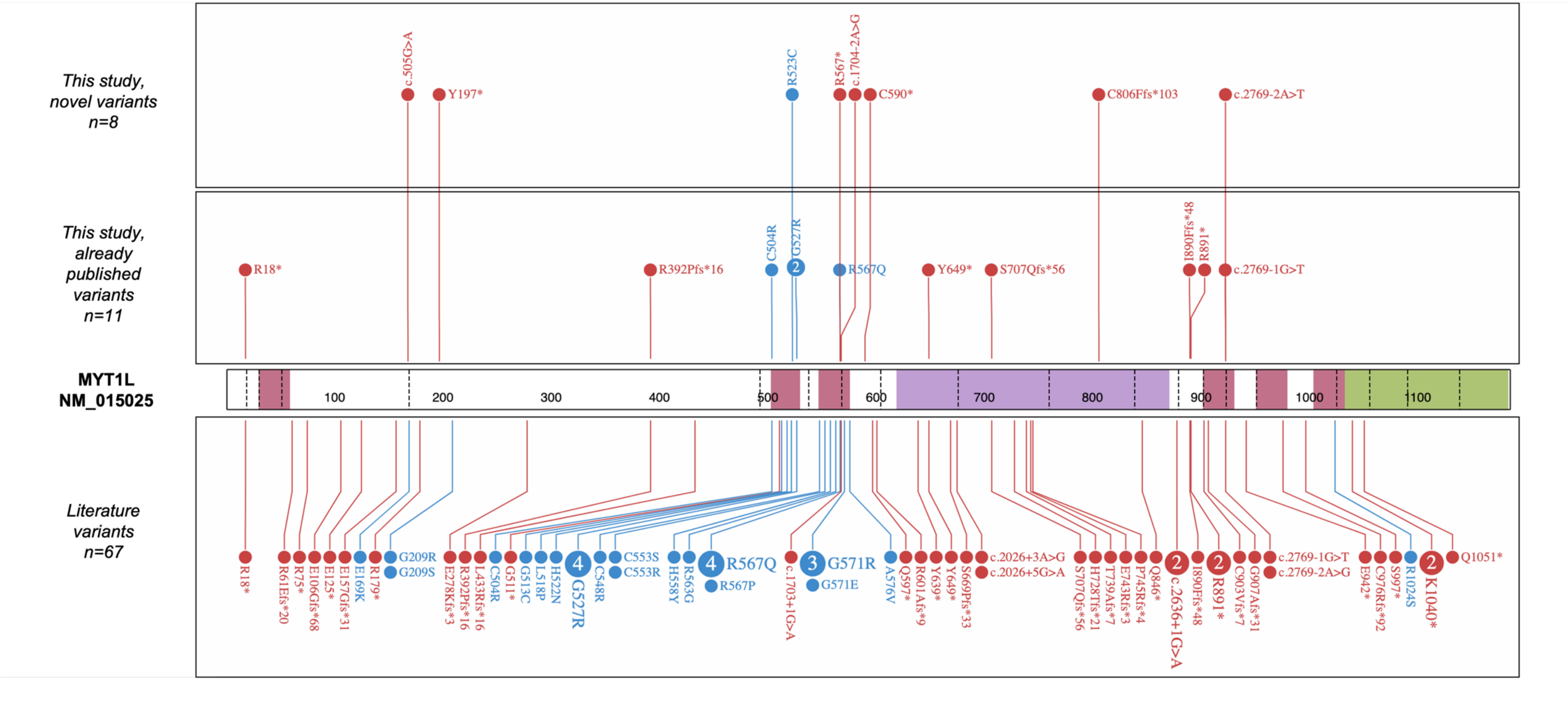

Supplementary Figure 1. Location of variants identified in this cohort and in previously published studies.
The representation is based on ProteinPaint (https://proteinpaint.stjude.org/). Zinc finger domains are represented in pink. The MYT1 domain is represented in purple. The Smc domain is represented in green. Variants from the current cohort are shown in the upper panel, divided into two subrectangles : novel variants appear at the top, previously reported ones below. Variants from the literature are displayed in the lower panel. MYT1L gene deletions are not included.

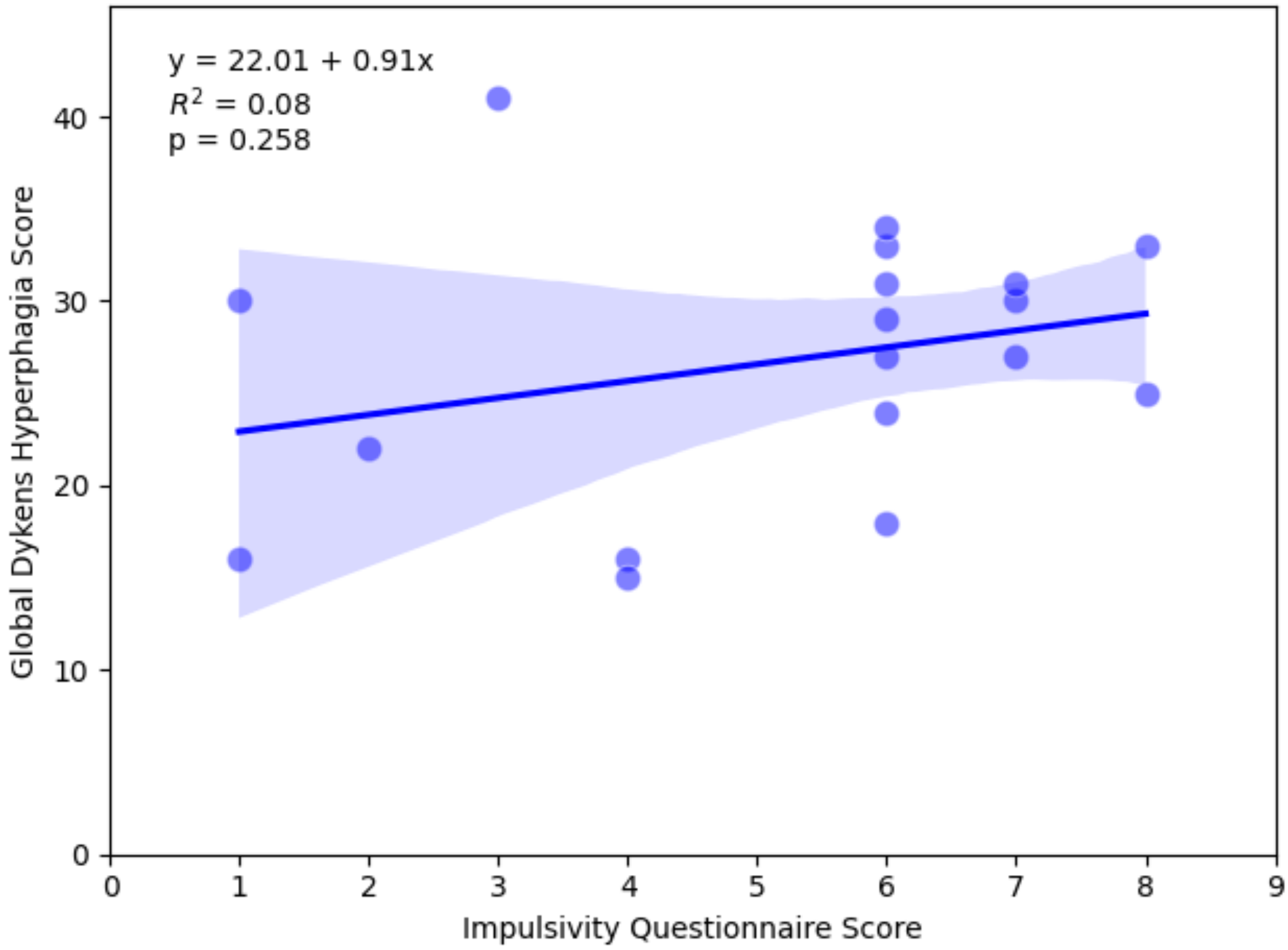

Supplementary Figure 2. Correlation between food-related impulsivity (Trousseau Questionnaire) and hyperphagia (Dykens' Hyperphagia Questionnaire).
No correlation can be observed between the high-impulsivity (≥ 7) and low-impulsivity (< 7) groups across the Dykens hyperphagia total score in the MYT1L cohort of 18 individuals.
